# How Old Is Too Old? Outcomes of Prolapse Native-Tissue Repair through Uterosacral Suspension in Octogenarians

**DOI:** 10.3390/life14040433

**Published:** 2024-03-24

**Authors:** Marta Barba, Alice Cola, Desirèe De Vicari, Clarissa Costa, Silvia Volontè, Matteo Frigerio

**Affiliations:** Department of Gynecology, IRCCS San Gerardo dei Tintori, University of Milano-Bicocca, 20900 Monza, Italy; m.barba8792@gmail.com (M.B.); d.devicari@campus.unimib.it (D.D.V.); c.costa14@campus.unimib.it (C.C.); s.volonte6@campus.unimib.it (S.V.)

**Keywords:** pelvic organ prolapse, native-tissue repair, quality of life, elderly population, fragility, complications

## Abstract

Introduction: Medical advancements are expected to lead to a substantial increase in the population of women aged 80 and older by 2050. Consequently, a significant number of individuals undergoing corrective prolapse surgery will fall into the elderly-patient category. The research indicates a notable rise in complications associated with prolapse surgery in patients older than 80, irrespective of frailty and other risk factors. Despite these challenges, the vaginal approach has been identified as the safest surgical method for pelvic organ prolapse (POP) repair in the elderly population. For this reason, we aimed to investigate the efficacy, complication rate, and functional outcomes associated with vaginal hysterectomy and an apical suspension/high uterosacral ligaments suspension as a primary technique for prolapse repair, both within a cohort of elderly patients. Methods: We retrospectively analyzed patients who underwent transvaginal hysterectomy plus an apical suspension procedure for stage ≥ II and symptomatic genital prolapse between January 2006 and December 2013. Anatomical and functional outcomes were evaluated. The Patient Global Impression of Improvement (PGI-I) score was used to evaluate subjective satisfaction after surgery. Results: Sixty-five patients were included in the analysis. The median age was 81.3 years. All individuals exhibited an anterior compartment prolapse stage II or higher, and the majority also a central prolapse stage II or higher. Notably, all participants reported symptoms of vaginal bulging. Over half of the population (58.6%) complained of incomplete bladder emptying. The intervention for all participants involved a vaginal hysterectomy with an apical suspension. Sixty-three patients (96.9%) and forty-four patients (67.6%) underwent a simultaneous anterior or posterior repair, respectively. Long-term complications (>30 days from surgery) were observed during follow-up, with a median duration of 23 ± 20 months. Seven (10.7%) anatomical recurrences were recorded, five (7.69%) concerning the anterior compartment, one (1.5%) the central, and three (4.6%) the posterior. Nevertheless, none of them necessitated further surgical intervention due to symptoms. Significant anatomical improvements for the anterior, central, and posterior compartments were noticed, compared to preoperative assessment (*p* < 0.001 for Aa and Ba, *p* < 0.001 for Ap and Bp, and *p* < 0.001 for C). PGI-I values established that 100% of patients were satisfied (PGI-I ≥ 2), with a median score of 1.12. Consequently, objective and subjective cure rates were 89.5% and 100%, respectively. Conclusion: Vaginal hysterectomy combined with apical suspension, particularly high uterosacral ligaments suspension, is a safe and effective primary surgical approach, even in elderly patients.

## 1. Introduction

Pelvic organ prolapse (POP) is a troublesome clinical condition defined by the herniation of at least one of the pelvic organs, such as the uterus, bladder, or rectum. The descent or bulge into the vaginal space arises from the weakened or damaged pelvic floor muscles and connective tissues. The pelvic floor acts like a sling, supporting these organs and maintaining their proper position within the pelvis. When this support system dwindles, it can lead to the displacement of one or more pelvic organs, resulting in pelvic organ prolapse. Determining the precise prevalence of pelvic organ prolapse is challenging. Discrepancies exist in the literature regarding the true prevalence of POP, and these variations may be associated with the type of study conducted. Indeed, the documented prevalence shows considerable variation across studies, with figures ranging from 3% to 50% [1]. Furthermore, it is imperative to take into consideration that the number of women with pelvic organ prolapse (POP) who do not seek medical attention is uncertain [2]. Moreover, the healthcare consequences of prolapse are expected to grow, due to the projections indicating a rising prevalence among the expanding population of elderly women [3]. The diagnosis of pelvic organ prolapse involves a comprehensive review of medical history, a thorough physical examination, and, occasionally, the use of imaging studies [4]. The extent of prolapse is assessed using the Pelvic Organ Prolapse Quantitation system (POP-Q), introduced in 1996 and endorsed by prominent organizations such as ICS, AUGS, SGS, and ACOG [5,6]. Although pelvic organ prolapse (POP) may not consistently manifest noticeable symptoms, typical indicators encompass sensations of fullness or pressure in the pelvic region, discomfort during sexual intercourse, and challenges with bowel movements or urination. Evaluation of symptoms is crucial, as treatment is typically not recommended for asymptomatic POP [7,8,9].

Obtaining a comprehensive medical history to identify symptoms associated with prolapse is crucial, including issues related to urinary, defecatory, and sexual functions [10]. Stress urinary incontinence (SUI) symptoms often coincide with stage I or stage II prolapse, and as prolapse advances, women may experience improvement in SUI but increased voiding symptoms [11,12,13,14]. Defecatory symptoms are more prevalent in women with POP compared to the general population, affecting 20% of women without POP and 24 to 52% of those with POP. Two of the symptoms most commonly associated with prolapse are constipation and incomplete emptying [12,15]. Moreover, the assessment of POP symptoms and their influence on a patient’s quality of life aids both patients and clinicians in establishing the treatment objectives [16,17,18].

The spectrum of treatment options ranges from conservative measures like pelvic floor exercises and lifestyle changes to more invasive interventions such as surgery. The choice of treatment depends on factors like symptom severity, impact on quality of life, and overall health. Surgical candidates for POP repair are symptomatic women for whom conservative management has failed or been declined. Surgical modality selection requires careful consideration, weighing obliterative versus reconstructive approaches. Reconstructive surgery for POP involves procedures targeting vaginal apex resuspension and correction of vaginal walls. The determination of the primary surgical approach for individuals with POP is contingent upon various factors, including overall health status, and the patient’s preferences. It is estimated that between 11 to 19% of females undergo surgery for prolapse or incontinence by the age of 80 to 85 years, with 30% of these individuals potentially requiring additional prolapse repair procedures [14,19,20]. Annually, in the United States, an estimated 200,000 inpatient surgical procedures for prolapse are undertaken [21].

It should also be taken into account that with advances in medical science there will be a significant tripling of the population of women aged 80 and older by 2050 [22]. Therefore, a large proportion of patients who undergo and will undergo corrective prolapse surgery will be in the elderly-patient category. Consequently, the choice among various surgical approaches must take into account the specific characteristics and peculiarities of this segment of the population. It was shown that the number of significant complications related to prolapse surgery shows a considerable increase within the demographic of patients older than 80 years, regardless of frailty or medical or surgical risk factors [23].

The approximate rate of severe complications stands at around 9.0% to 25% among the very-elderly population. Notably, individuals in this age group face elevated risks of cardiac complications, stroke, and mortality. Certain specific complications, including the need for blood transfusion or the experiencing of significant blood loss, as well as occurrences like pulmonary edema and postoperative congestive heart failure, have been found to be correlated with various risk factors. These risk factors encompass aspects such as the duration of surgery, the presence of coronary artery disease, and the existence of peripheral vascular disease [24].

Furthermore, it is noteworthy to mention that the prevalence of acute postoperative urinary retention (POUR) subsequent to pelvic reconstructive surgery, impacting approximately 15–45% of women, does not appear to exhibit any correlation with frailty among older women undergoing prolapse surgery. This suggests that the occurrence of POUR in this context is not influenced by the frailty status of older female patients undergoing prolapse surgery [25].

The vaginal approach has been demonstrated to be the safest overall surgical approach for POP repair in the elderly population [26]. Currently, there is a renewed interest among scholars in native-tissue techniques for pelvic organ prolapse repair, primarily driven by considerations of cost-effectiveness and the avoidance of complications associated with mesh application. Within the context of native-tissue apical procedures, the suspension of the high uterosacral ligaments (USLs) is acknowledged as a credible and effective choice for central compartment repair. However, existing research on uterosacral ligaments has often been limited, with small participant cohorts or insufficient follow-up durations.

Consequently, our objective is to assess the efficacy, complication rate, and functional outcomes associated with vaginal hysterectomy and an apical suspension/high uterosacral ligaments suspension as a primary technique for prolapse repair within a cohort of elderly patients.

## 2. Materials and Methods

This is a retrospective single-center study analyzing data from patients undergoing vaginal hysterectomy followed by high uterosacral ligaments suspension for POP between January 2006 and December 2013 in the gynecology unit of San Gerardo Hospital in Monza. Only women aged 80 years or more at the time of surgery were considered.

The preoperative assessment involved a medical interview to evaluate obstetrical history. The presence of urinary, sexual, and bowel symptoms was determined based on the standardized terminology of the International UroGynecology Association and the International Continence Society [27]. For each patient, a urogenital examination was performed, and genital prolapse was staged in accordance with the Pelvic Organ Prolapse Quantification (POP-Q) system [5].

**Figure 1:**
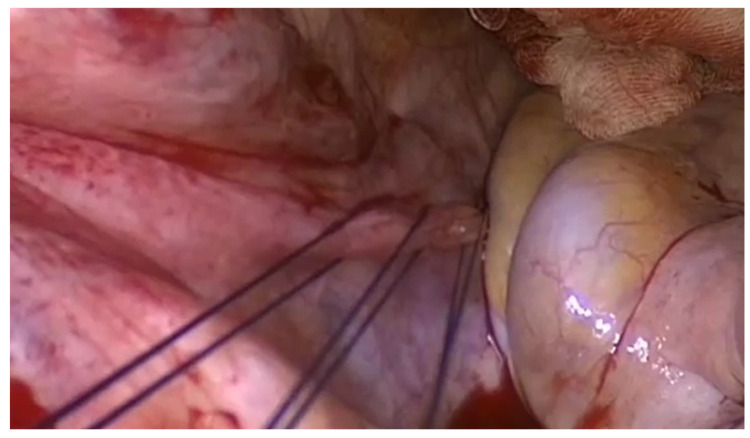
High uterosacral ligament transfixion—right side. Image was acquired for didactic purposes through a laparoscopic camera by vaginal route to better demonstrate the USL transfixion.

Patients underwent transvaginal hysterectomy and salpingectomy, with bilateral oophorectomy performed when technically feasible, based on factors such as menopausal status, age, oncologic risk, and patient preferences. All surgeries were carried out by experienced pelvic floor surgeons. Following vaginal hysterectomy, an apical suspension procedure was performed. The high uterosacral ligament (USL) suspension was carried out using two or three monofilament, absorbable, size 0 sutures per side, as previously described [28]. Stitches were positioned at the level of ischial spines or above (Figure 1). Additional surgical procedures, such as anterior and/or posterior repair, were undertaken as necessary. Specifically, anterior repair involved midline fascia plication with non-absorbable interrupted sutures from the level of the bladder neck to the apex of the anterior vaginal wall, with the duplicated fascia apex incorporated using suspension sutures [29,30]. Posterior repair was performed as native-tissue procedure using the technique previously described [31]. A posterior colpotomy, shaped like an inverted-T and extending from the vaginal apex to the hymen, was executed. Thorough dissection of the posterior vaginal wall from the rectovaginal tissue was conducted to isolate rectal prolapse, and reduction was achieved through half-purse string sutures. Identification of the puborectalis muscle, located laterally to the rectum and distal from the ischial spines, was performed. Direct vision was employed to place midline plication sutures on the most medial part of the puborectalis muscles on each side. The procedure was completed with perineoplasty and posterior colporrhaphy. The patients underwent follow-up examinations at 1 month and 1 year post-surgery, followed by yearly visits. These follow-ups consisted of a clinical interview and a thorough urogenital examination. A subjective recurrence after surgery was defined by the presence of bulging symptoms postoperatively. Objective recurrence was determined by a postoperative descent to stage II or below according to the POP-Q system, or the necessity for a reoperation. The evaluation of subjective satisfaction post-surgery utilized the Patient Global Impression of Improvement (PGI-I) score. Quality of Life (QoL) success was delineated by responses indicating “very much improved” or “much improved” on the PGI-I score (score ≤ 2). For statistical analysis, we considered symptoms, POP-Q evaluation, and QoL data at the last available follow-up.

This study received approval from the Institutional Review Board of San Gerardo Hospital in Monza, Italy (SH-MCC 1709/2013). Data acquisition involved the utilization of hospital-dedicated software for clinical monitoring of patients. The data entry process was conducted by one author, with a subsequent verification by another author to ensure accuracy. Descriptive statistics were computed as absolute numbers, with percentages for categorical variables and mean plus or minus the standard deviation for continuous variables. The assessment of differences involved paired *t*-tests for continuous data and Fisher’s test for noncontinuous data. Statistical analysis was executed using JMP 17.2 (SAS Institute located in Cary, NC, USA). A *p*-value < 0.05 was deemed statistically significant.

## 3. Results

In the specified interval, a total of 67 patients underwent vaginal hysterectomy and received an apical suspension high USL suspension in accordance with the predefined inclusion criteria. However, two individuals were omitted from the analysis because of either incomplete data or loss to follow-up within the specified timeframe. Consequently, the analysis was based on the evaluation of 65 patients.

The characteristics of the study population are comprehensively outlined in Table 1. The median age of the participants was 81.3 years. The mean age of onset of menopause was 50.7. Only two participants were sexually active, and only two had undergone hormone replacement therapy. Baseline symptoms and POP-Q metrics are detailed in Table 2. All individuals exhibited an anterior compartment prolapse stage II or higher, and the majority also a central prolapse stage II or higher. Notably, all participants reported symptoms of vaginal bulging. Over half of the population (58.6%) complained of incomplete bladder emptying. Among other urinary disorders, 17 (26.1%) patients complained of stress urinary incontinence, 21 (32.3%) of overactive bladder syndrome, and 19 of urge incontinence (29.2%).

The intervention for all participants involved a vaginal hysterectomy with an apical suspension. Severally, 63 patients (96.9%) and 44 patients (67.6%) underwent a simultaneous anterior or posterior repair, respectively (Table 3). The interventions were completed with a median operative time of 100.8 ± 25.94 min and a median blood loss of 243.8 ± 149.1 mL. Eight patients incurred a total blood loss equal to or surpassing 500 mL, while no other intraoperative complications were noted. Throughout the designated monitoring interval, there were no instances of prolonged postoperative issues observed beyond the 30-day threshold, with a median follow-up duration of 23 ± 20 months. Seven (10.7%) anatomical recurrences were recorded, five (7.69%) concerning the anterior compartment, one (1.5%) the central, and three (4.6%) the posterior (Table 4). Nevertheless, none of them necessitated further surgical intervention due to symptoms. Consequently, objective and subjective cure rates were 89.5% and 100%, respectively. The comparison of preoperative and postoperative vaginal profiles based on the POP-Q system is presented in Table 2, revealing significant improvements for the anterior (*p* < 0.001 for Aa and Ba), central (*p* < 0.001 for C), and posterior compartments (*p* < 0.001 for Ap and Bp). Functional outcomes, as outlined in Table 4, demonstrated significant reductions in constipation and vaginal bulging, compared to baseline (*p* < 0.001). Quality-of-life assessments utilizing PGI-I indicated that 100% of patients were satisfied, with fifty-seven (87.6%) patients defining themselves as very much better and eight (12.3%) as much better (median PGI-I score stood at 1.12; 1.0 ≤ IQR ≤ 2.0).

## 4. Discussion

The decision-making process prior to pelvic organ prolapse surgery poses complexities for both patients and physicians. Several considerations and communications are involved, including those regarding the elective nature of the procedure, non-surgical management options, potential perioperative surgical and medical complications, and specific risks like prolapse recurrence, de novo lower urinary tract symptoms, and complications related to mesh usage. This counselling complexity is particularly pronounced when dealing with elderly patients. One of the primary considerations influencing the selection of treatment in this population is the risk of complications; as demonstrated by Chapman et al., the category of individuals aged more than 80 years, “very-elderly” persons, continued to demonstrate an independent association with serious complications (adjusted odds ratio [aOR] 2.01, 95% CI 1.8–2.3) [23].

For this reason, if surgical therapy is deemed a viable option, the choice among various approaches becomes of paramount importance in this type of population. When Yavad et al. assessed vaginal colpopexy against all alternative routes, such as minimally invasive sacrocolpopexy and minimally invasive sacrocolpopexy, the technique exhibited reduced incidence of life-threatening complications, readmissions, mortality within 30 days, pneumonia, and stroke [28]. Although recent data have demonstrated enduring and positive functional outcomes in transvaginal native-tissue repair for pelvic organ prolapse during long-term follow-up [32], there is still a notable absence of information regarding the efficacy of this specific surgical method for the elderly population.

Consequently, the primary objective of this study is to examine not only the safety of this specific surgical procedure in a population more prone to complications but also to evaluate its efficacy in terms of specific outcomes for this demographic. We observed that the transvaginal USL suspension is a durable, efficient, and secure procedure in elderly patients, contributing positively to functional outcomes. Within our study population, we achieved notable subjective cure rates of 89.5% and objective cure rates of 100%. Anatomical recurrence was minimal, at 10.7%, and reoperation was not necessary in these cases. All patients reported a self-perceived improvement, ranging from good to very good. Additionally, positive effects on constipation, bulging, and voiding symptoms were evident. In particular, voiding symptoms drastically decreased from 58.4% at baseline to 12.3% after surgery.

The recent increase in the adoption of native-tissue repair procedures for POP comes in the wake of warnings from the US Food and Drug Administration (FDA) concerning the safety and effectiveness of synthetic meshes. This surge underscores the necessity for a thorough re-examination of these procedures and emphasizes the importance of reassessing native-tissue repair methods. Furthermore, it accentuates the critical need for a concerted effort to systematically document and report the long-term outcomes associated with these techniques. Recently, good long-term outcomes of native-tissue procedures have been reported for high USL.

In a retrospective study conducted by Deo et al., a thorough analysis of a 5-year follow-up after the repair of primary prolapse through high uterosacral ligament suspension was performed. The study revealed that long-term outcomes following high uterosacral ligament suspension were deemed satisfactory, with subjective and objective cure rates of 85% and 87%, respectively [33]. Additionally, the reoperation rate was reported to be 1.1%. Subsequently, that study’s findings were validated by Cola et al. after an additional 5-year period. Their study demonstrated consistently favorable outcomes, with subjective and objective cure rates of 93.9% and 80.9%, respectively, along with a reoperation rate of 2.1% [32]. In a retrospective analysis by Duan et al., a cohort comprising 104 patients was examined nine years post-uterosacral ligament (USL) suspension. The study documented a noteworthy 91% cure rate, defined by the absence of prolapse beyond the hymen, the absence of prolapse symptoms, and the lack of requirement for reoperation or pessary use. The recurrence of pelvic organ prolapse (POP) beyond the hymen was predominantly observed in the anterior compartment (6.7%), followed by the posterior compartment (2.9%) [34]. Similarly, in a retrospective evaluation by Schiavi et al., encompassing 414 patients who underwent either modified McCall or Shull suspension, anatomical failure was reported in 21.0% of cases after a median follow-up of 8.9 years. The anterior compartment exhibited the highest incidence of anatomical failure (12.8%). Prolapse symptoms were reported by 9.4% of women, and 1.5% necessitated reoperation due to the recurrence of prolapse [35].

The recurrence patterns observed after USL suspension, as delineated in these studies, demonstrated reproducibility and consistency with our own series. Notably, the anterior compartment emerged as the most common site of recurrence, whereas apical relapse was found to be infrequent following USL suspension.

Aligning with our findings, a prevalent observation across these studies was that most women experiencing anatomical recurrences were asymptomatic.

In contrast to the findings in the aforementioned studies, none of our patients reported a recurrence of bulging symptoms, obviating the need for reintervention. This divergence may be rationalized by the observation that these recurrences are often of modest severity. Consequently, it can be inferred that with comparable anatomical outcomes, older patients may experience a lower intensity of symptoms, potentially rendering the same prolapse asymptomatic. This underscores the nuanced interplay between anatomical outcomes and symptomatic manifestation, suggesting that the impact on quality of life may vary, particularly in older individuals.

The commendable Patient Global Impression of Improvement (PGI-I) scores within our patient cohort appear to substantiate this hypothesis.

While the overall life intensity of older patients may indeed be lower compared to the general population, it is crucial to recognize that aging is intricately linked to various disorders. Conditions such as pelvic organ prolapse, lower urinary tract symptoms, dysfunctional bowel and bladder evacuation, and sexual dysfunction require attention [36].

Silva et al. conducted an evaluation of 5-year functional outcomes following USL suspension for uterovaginal or vaginal vault prolapse repair. Their study documented a sustained positive influence on bladder function, evidenced by postoperative incontinence impact questionnaire/urogenital distress inventory scores, showcasing significant improvement across all domains (irritative, obstructive, and stress), compared to preoperative measures. Additionally, in sexually active women, 94% expressed satisfaction with their sexual activity post-surgery, despite abnormal FSFI desire scores [37]. In our prior analysis, we emphasized the noteworthy outcomes observed in a sample of 28 patients who underwent repair for isolated rectocele using native tissue through a transvaginal approach. The results indicated a significant improvement, both anatomically (*p* < 0.001 for Ap and Bp) and functionally. This improvement was manifested by a reduction in vaginal bulging and a mitigation of obstructed-defecation symptoms [38].

Similarly, in our own series, a consistent positive impact on urinary and bowel function was observed, manifesting as reductions in constipation and voiding symptoms. Moreover, despite the significant aging of patients, notably, there was an observed rise in sexually active women (two vs. four), though this was not statistically significant. Nonetheless, this may serve as an indirect indication of an improved quality of life.

Consequently, based on our findings, and corroborating evidence from previous reports, it can be concluded that vaginal hysterectomy and apical suspension, particularly employing high uterosacral ligament suspension, constitutes an effective procedure for achieving anatomical restoration and alleviating symptoms associated with pelvic organ prolapse. Notably, this approach is also characterized by a high safety profile. It is pertinent to acknowledge that, while mesh repair surgery exhibits higher rates of anatomical cure and patient satisfaction, it is accompanied by elevated risks of post-surgical and late complications when compared to native-tissue repair (NTR). Specifically, in a comprehensive analysis conducted by Capobianco et al., patients undergoing anterior compartment NTR demonstrated lower incidence of de novo stress, urinary incontinence, and musculoskeletal pain [39].

As per Margulies et al.’s meta-analysis, the primary concern among the specific complications associated with the uterosacral ligament suspension procedure is ureteral injury, occurring at an average rate of 1.8%. There is also a 1.3% incidence of patients requiring a subsequent hemotransfusion [40]. Notably, in the over-80-year-old population, the rate of hemotransfusion requirement increases to 2.6%, which is particularly increased by subtle intraoperative blood loss [25]. It is noteworthy that none of these complications were reported within our patient population. Furthermore, only 12.3% of patients experienced a blood loss equal to or exceeding 500 mL, and, notably, in no instance did this necessitate hemotransfusion.

To our knowledge, this study stands as the inaugural investigation focusing on anatomical, functional, and quality-of-life outcomes in a highly specific subset of patients aged over 80 years. The cohort underwent vaginal hysterectomy and apical suspension specifically employing high uterosacral ligaments suspension for the management of pelvic organ prolapse (POP). Significantly, our assessment encompassed an extended follow-up period, thereby contributing valuable insights into the enduring effects of these procedures within the context of an elderly demographic.

The limitations of the study include its retrospective design, the limited follow-up, and the constrained number of patients subjected to analysis. Nevertheless, a deliberate decision was made to exclusively assess patients considered very-elderly. This decision was undertaken to systematically investigate a specific subpopulation of patients undergoing surgical treatment for prolapse, with the objective of elucidating their distinctive characteristics and requirements. Another limitation is the lack of a dedicated QoL questionnaire in this study apart from the PGI-I.

## 5. Conclusions

In conclusion, vaginal hysterectomy and an apical suspension/high uterosacral ligaments suspension constitutes a secure and efficacious primary surgical modality, even in elderly patients, and should be considered a valid approach for women who desire to preserve sexual activity.

## Figures and Tables

**Table 1:** Patients’ characteristics. Continuous data as mean ± standard deviation; non-continuous data as absolute numbers with percentages.

Mean age	81.3 ± 1.9
BMI (kg/m^2^)	24.7 ± 3.6
Parity	2.2 ± 1.0
Maximum child weight (g)	3637.4 ± 562.1
Menopause	65 (100%)
Mean age of menopause onset	50.7 ± 3.6
Sexually active	2 (3.07%)

**Table 2:** Baseline and outcomes data. Continuous data as mean ± standard deviation; non-continuous data as absolute numbers with percentages. Aa: fixed point, midline of anterior vaginal wall, located 3 cm proximal to external urethral meatus; Ba: moving point, most distal portion of any part of anterior vaginal wall; C: leading edge of vaginal cuff (post-hysterectomy); gh: genital hiatus; pb: perineal body; tvl: total vaginal length; Ap: fixed point, midline of posterior vaginal wall, located 3 cm proximal to hymen; Bp: moving point, most distal portion of any part of posterior vaginal wall.

	Preoperative	Postoperative	*p*-Value
Aa	+1.6 ± 1.3	−2.3 ± 0.8	<0.0001
Ba	+1.9 ± 1.4	−2.4 ± 0.8	<0.0001
C	+0.9± 2.5	−6.9 ± 2.0	<0.0001
Gh	3.6 ± 0.6	3.2 ± 0.7	0.0002
Pb	2.9 ± 0.4	2.8 ± 0.4	0.1522
Tvl	9.9 ± 1.2	8.4 ± 0.4	<0.0001
Ap	−1.2 ± 1.6	−2.7 ± 0.6	<0.0001
Bp	−1.2± 1.7	−2.7 ± 0.6	<0.0001
D	−3.9 ± 2.9	/	N/A
Stress urinary incontinence	17 (26.1%)	12 (18.4%)	0.2786
Overactive bladder syndrome	21 (32.3%)	14 (21.5%)	0.1276
Urge urinary incontinence	19 (29.2%)	7 (10.7%)	0.0094
Voiding symptoms	38 (58.4%)	8 (12.3%)	<0.0001
Sexual activity	2 (3.7%)	6 (9.2%)	0.0832
Constipation	17 (26.1%)	11 (16.9%)	<0.0001
Bulging symptoms	65 (100%)	0	<0.0001

**Table 3:** Operative data. Continuous data as mean ± standard deviation; non-continuous data as absolute numbers with percentages.

Vaginal hysterectomy	65 (100%)
Uterosacral ligaments suspension	65 (100%)
Anterior repair	63 (96.9%)
Posterior repair	44 (67.6%)
Mean blood loss	243.8 ± 149.1
Blood loss ≥ 500 mL	8 (12.3%)
Mean operative time in minutes	100.8 ± 25.9

**Table 4:** Objective outcomes. Continuous data as mean ± standard deviation; non-continuous data as absolute numbers with percentages.

Anatomic recurrence	7 (10.7%)
Anterior compartment relapse	5 (7.7%)
Central compartment relapse	1 (1.5%)
Posterior compartment relapse	3 (4.6%)
Mean follow-up time (months)	23 ± 20
Reoperation	0

## Data Availability

The data presented in this study are available on request from the corresponding author.
